# Older Adults’ Views on Insurance Coverage for Weight Management Medications

**DOI:** 10.1001/jamanetworkopen.2025.2008

**Published:** 2025-03-26

**Authors:** Lauren Oshman, Matthias Kirch, Erica Solway, Dianne C. Singer, Preeti N. Malani, J. Scott Roberts, Jeffrey T. Kullgren, Dina Hafez Griauzde

**Affiliations:** 1 Department of Family Medicine, University of Michigan, Ann Arbor; 2 Institute for Healthcare Policy and Innovation, University of Michigan, Ann Arbor; 3 Department of Internal Medicine, Division of Infectious Diseases, University of Michigan, Ann Arbor; 4 Department of Health Management and Policy, University of Michigan, Ann Arbor; 5 Department of Health Behavior and Health Equity, School of Public Health, University of Michigan, Ann Arbor; 6 Center for Clinical Management Research, VA Ann Arbor Healthcare System, Ann Arbor, Michigan; 7 Department of Internal Medicine, University of Michigan, Ann Arbor; 8 VA Ann Arbor Healthcare System, Ann Arbor, Michigan

## Abstract

**Importance:**

Medicare and many commercial insurers do not cover US Food and Drug Administration–approved weight management medications, such as incretin mimetics (semaglutide, tirzepatide, and liraglutide), bupropion-naltrexone, and phentermine-topiramate).

**Objectives:**

To assess older adults’ interest in weight management medications and associated characteristics and to understand their perceptions about insurance coverage of weight management medications.

**Design, Setting, and Participants:**

This survey study used cross-sectional data from a nationally representative sample of US adults ages 50 to 80 years from the July 2023 National Poll on Healthy Aging. Data were analyzed from August to November 2023.

**Main Outcomes and Measures:**

Outcomes of interest were estimates of interest in taking weight management medication and perceptions about coverage for US Food and Drug Administration–approved weight management medications by insurers, including Medicare.

**Results:**

Among 2657 respondents, 60.3% (95% CI, 56.7%-63.8%) were ages 50 to 64 years, 52.2% (95% CI, 49.8%-54.5%) were female, and 10.6% (95% CI, 9.3%-12.0%) were non-Hispanic Black, 11.4% (95% CI, 10.4%-12.6%) were Hispanic, and 70.3% (95% CI, 68.2%-72.4%) were non-Hispanic White. Overall, 35.1% (95% CI, 31.9%-38.4%) of participants were interested in using weight management medications, including 59.1% (95% CI, 53.4%-64.5%) of individuals with body mass index (BMI) of 30 or greater. Interest was most robustly associated with having used these medications in the past (adjusted odds ratio, 7.57 [95% CI, 4.41-13.02]) and BMI of 30 or greater (adjusted odds ratio, 5.04 [95% CI, 3.48-7.30]). Most participants (of any BMI) agreed that health insurance should cover weight management medications (2176 of 2625 respondents [83.2%]). When asked whether Medicare should cover such medications, most still favored coverage (2097 of 2616 respondents [75.7%]), but fewer approved of paying more for a Medicare premium to ensure coverage (829 of 2604 respondents [30.2%]).

**Conclusions and Relevance:**

In this survey study of older US adults, most participants agreed that Medicare should cover weight management medications and more than half of those with BMI of 30 or greater were interested in using them. These results should inform decisions to include weight management medications in the Medicare and commercial insurance programs, as well as utilization policies to control health care costs.

## Key Points

**Question:**

Are older adults interested in using weight management medications to treat obesity, and do they believe Medicare should cover weight management medications?

**Findings:**

In this survey study of 2657 US adults ages 50 to 80 years, 35.1% of participants overall and 59.1% of those with body mass index of 30 or greater were interested in taking weight management medications. Most respondents (75.7%) favored Medicare coverage, but only 30.2% approved of paying more in Medicare premiums to ensure coverage.

**Meaning:**

These findings suggest that older adults’ high interest in use of weight management medications should be taken under consideration by Medicare policymakers.

## Teaser

This survey study assesses older US adults’ perspectives on use of and insurance coverage for weight management medications and examines characteristics associated with support for coverage by insurers, including Medicare.

## Introduction

Obesity is one of the most prevalent chronic diseases in the US, with approximately half of all adults estimated to have obesity by 2030.^1^ Obesity is also a risk factor for many costly medical conditions, including type 2 diabetes, cardiovascular disease, kidney and liver disease, and cancer.^2^ Obesity was associated with an estimated $173 billion in US annual health care spending per year in 2019 dollars, with the highest estimated costs occurring among people ages 60 to 70 years with severe obesity.^3^ Overall social and economic costs of obesity were estimated at $1.2 trillion, or 6.7% of gross domestic product, in 2018 dollars.^4^ Even modest reduction in body weight of 5% can improve health outcomes and reduce health care costs through a variety of effective treatments, including lifestyle change, weight management medication (WMM), and metabolic surgery.^5,6^

Since 2012, new and more effective WMMs have become available for treatment of obesity or overweight with comorbidities, such as hypertension or obstructive sleep apnea. The newest class of medications, incretin mimetics, includes glucagon-like peptide-1 and gastric inhibitory polypeptide receptor agonists. To date, the US Food and Drug Administration (FDA) has approved 3 medications in this class for weight management: liraglutide in 2014, semaglutide in 2021, and tirzepatide in 2023.^7^ These medications are effective at treating obesity and preventing and treating complications of obesity, including obstructive sleep apnea, cardiovascular disease, chronic kidney disease, and diabetes.^8,9^ Older WMMs, including phentermine, approved in 1959; phentermine-topiramate, approved in 2012; and bupropion-naltrexone, approved in 2014, are also approved by the FDA for the treatment of obesity or overweight with comorbidities.^10^

A historical statutory exclusion prevents Medicare Part D from covering “agents when used for anorexia, weight loss, or weight gain,” although Medicare Part D now covers 1 incretin mimetic, semaglutide, for secondary prevention of cardiovascular disease among individuals with prior myocardial infarction, stroke, or peripheral arterial disease.^11,12^ The Biden administration proposed a rule requiring Medicare to cover WMMs for obesity, rather than specific obesity-related complications.^13^ The Treat and Reduce Obesity Act would require Medicare to cover WMMs for obesity as a disease and passed the US House of Representatives Ways and Means Committee in July 2024.^14^ The Congressional Budget Office estimated that covering WMMs would increase use among eligible beneficiaries (persons with obesity or overweight and a qualifying comorbidity) from 2% to 14% and increase federal spending by $35 billion over 8 years.^15^ Other estimates of the effect of coverage on Medicare spending are widely variable and fluctuate based on assumptions of drug costs, rates of uptake, adherence to treatment, and downstream changes in spending on obesity complications. One study suggested that coverage of semaglutide for 10% of Medicare beneficiaries with obesity could cost $26.8 billion, or 18% of Medicare Part D net spending.^16^ Another study estimated that coverage of incretin mimetics for 10% of eligible Medicare beneficiaries could lead to an increase in spending of only $6.1 billion.^17^ At present, approximately one-third of US employers’ commercial insurers cover WMMs, placing older adults at risk for losing WMM coverage when they transition from employer-sponsored to Medicare insurance.^18^

While a 2024 Kaiser Family Foundation poll demonstrated positive public opinion in the general US adult population regarding Medicare coverage of incretin mimetics for weight management,^19^ little is known about older adults’ interest in using WMM and perceptions about Medicare coverage, which could increase premiums. To address this gap, we used the National Poll on Healthy Aging to survey older adults about their perspectives on the use of and insurance coverage for WMM. The aim of this study is to characterize older adults’ interest in using WMM and associated characteristics and their perceptions about insurance coverage for WMM.

## Methods

This survey study was deemed exempt from review and informed consent by the University of Michigan institutional review board because data were deidentified. This study is reported following the American Association for Public Opinion Research (AAPOR) reporting guideline.

### Study Design

This study is a primary analysis of data from a cross-sectional survey conducted in partnership with the University of Michigan National Poll on Healthy Aging (NPHA). The NPHA is directed by the University of Michigan Institute for Healthcare Policy and Innovation, sponsored by AARP and Michigan Medicine, and conducted using the AmeriSpeak Panel by NORC at the University of Chicago. The NPHA is fielded twice a year and uses a probability-based sample of adults ages 50 to 80 years designed to be nationally representative of the US household population. The NPHA examines the experiences and perspectives of middle-aged and older adults on a variety of health topics.

### Participant Sample

The survey module was administered online and via phone from July 17 to August 7, 2023, to a randomly selected, stratified group of US adults ages 50 to 80 years. The sample was subsequently weighted to reflect population figures based on US Census Bureau data using TrueNorth Calibration weighting methods.^20^

### Study Measures and Variables

Primary outcomes were older adults’ interest in using WMM and perceptions about whether health insurance should cover FDA-approved WMMs for the treatment of overweight or obesity. Variables included participant-reported sociodemographic characteristics, including age, gender, race, ethnicity, income, employment status, and highest level of education. Participants also reported health status, health insurance literacy, and chronic health conditions (eg, hypertension, diabetes, heart disease), and anthropometric measures, including current weight and height and historical and current overweight status; we used the term *overweight* rather than *obesity* because this term was preferred in pilot presurvey testing. We calculated body mass index (BMI; calculated as weight in kilograms divided by height in meters squared) using reported weight and height. Survey questions specific to weight management treatment explored participants’ (1) knowledge about WMMs; (2) source of WMM information; (3) prior use of weight management treatment options, including diet and physical interventions, WMMs, and bariatric surgery; (4) interest in using WMM; (5) perceptions about whether health insurance should cover weight management treatments, including WMM; and (6) willingness to pay more for insurance coverage of WMM. Lastly, participants were asked whether obesity is a chronic condition (4-point Likert scale). Survey questions are available elsewhere.^21^

### Statistical Analysis

We summarized the frequency of responses with actual numbers and weighted percentages to key variables of interest and performed Pearson χ^2^ test to explore associations between the primary outcomes and variables of interest. We performed a logistic regression analysis to explore associations between participant interest in using WMM and their attitudes regarding insurance coverage adjusted for demographic variables, chronic health conditions, and experiences and attitudes about obesity. Two-sided *P* < .05 was considered statistically significant. We used Stata software version 18 (StataCorp) for all statistical analysis, and analysis was conducted from September 2023 to May 2024.

## Results

Of 5294 people invited, 2657 individuals (50.2%) responded and completed the survey, with 202 (7.6%) completing via phone and 2455 (92.4%) completing via web browser. Respondents were more likely than nonrespondents to be ages 65 to 80 years, non-Hispanic White, and have a college degree or higher. After weighing, the study sample included 60.3% (95% CI, 56.7%-63.8%) of participants ages 50 to 64 years and 52.2% (95% CI, 49.8%-54.5%) of participants were female. By race and ethnicity, 10.6% (95% CI, 9.3%-12.0%) were non-Hispanic Black, 11.4% (95% CI, 10.4%-12.6%) were Hispanic, and 70.3% (95% CI, 68.2%-72.4%) were non-Hispanic White. Regarding health conditions, 52.1% (95% CI, 49.8%-54.4%) had hypertension, 24.9% (95% CI, 21.3%-28.8%) had diabetes, and 13.1% (95% CI, 10.9%-15.8%) had heart disease. Additional characteristics are presented in Table 1 and stratified by BMI of 27 or greater vs less than 27, representing the BMI at which WMMs may be indicated for patients affected by overweight and comorbidities, such as hypertension, hyperlipidemia, or prediabetes.

**Table 1.** Characteristics of All Respondents and Stratified by BMI

Characteristic	% (95% CI)		
	Total	BMI^a^	
		<27	≥27
Age group, y			
50-64	60.3 (56.7-63.8)	56.5 (52.9-60.0)	63.4 (58.3-68.3)
65-80	39.7 (36.2-43.3)	43.5 (40.0-47.1)	36.6 (31.7-41.7)
Gender			
Male	47.8 (45.5-50.2)	40.6 (37.0-44.4)	53.6 (50.2-56.9)
Female	52.2 (49.8-54.5)	59.4 (55.6-63.0)	46.4 (43.1-49.8)
Calculated BMI category			
Underweight	1.6 (1.1-2.4)	3.8 (2.5-5.7)	NA
Healthy weight	26.1 (23.5-28.9)	61.1 (57.2-64.9)	NA
Overweight	36.5 (34.3-38.8)	35.1 (31.6-38.7)	37.5 (34.5-40.6)
Obesity	35.8 (33.1-38.6)	NA	62.5 (59.4-65.5)
Race and ethnicity			
Hispanic	11.4 (10.4-12.6)	9.4 (8.1-10.9)	13.1 (11.6-14.7)
Non-Hispanic Asian-Pacific Islander	5.6 (4.2-7.6)	7.9 (4.6-13.4)	3.8 (2.7-5.4)
Non-Hispanic Black	10.6 (9.3-12.0)	7.9 (6.4-9.7)	12.7 (10.4-15.6)
Non-Hispanic other race	1.0 (0.7-1.4)	1.2 (0.8-1.9)	0.8 (0.5-1.3)
Non-Hispanic multiple races	1.0 (0.7-1.4)	0.8 (0.4-1.8)	1.1 (0.7-1.8)
Non-Hispanic White	70.3 (68.2-72.4)	72.7 (67.9-77.1)	68.5 (65.4-71.3)
Education			
≤High school diploma	11.3 (9.6-13.3)	11.0 (8.2-14.6)	11.6 (10.2-13.2)
Some college	28.9 (26.4-31.5)	27.5 (23.5-32.0)	30.0 (27.4-32.7)
≥Bachelor’s degree	59.8 (56.3-63.2)	61.5 (56.0-66.8)	58.4 (55.2-61.5)
Annual household income, $			
<30 000	14.6 (13.0-16.4)	13.1 (10.3-16.7)	15.8 (13.4-18.6)
30 000 to <60 000	21.6 (19.9-23.4)	20.7 (18.2-23.5)	22.3 (19.8-25.1)
60 000 to <100 000	26.6 (24.5-28.7)	25.6 (22.4-29.1)	27.3 (23.5-31.5)
≥100 000e	37.2 (34.9-39.6)	40.6 (35.2-46.2)	34.5 (31.0-38.2)
Federal poverty level			
≥100%	92.4 (90.9-93.7)	93.8 (90.1-96.1)	91.4 (89.8-92.8)
<100%	7.6 (6.3-9.1)	6.2 (3.9-9.9)	8.6 (7.2-10.2)
Employment status			
Employed	54.0 (50.8-57.3)	50.6 (46.8-54.4)	56.8 (51.8-61.7)
Retired	33.8 (30.7-37.0)	38.7 (35.3-42.1)	29.9 (25.7-34.5)
Not working or receiving SSDI	12.2 (10.1-14.7)	10.7 (7.9-14.4)	13.3 (11.0-16.0)
Metropolitan area			
No	15.3 (13.6-17.1)	12.7 (10.7-14.9)	17.4 (14.8-20.4)
Yes	84.7 (82.9-86.4)	87.3 (85.1-89.3)	82.6 (79.6-85.2)
Health characteristics			
Hypertension or high blood pressure	52.1 (49.8-54.4)	36.2 (32.8-39.7)	64.8 (60.1-69.1)
Diabetes or high blood glucose	24.9 (21.3-28.8)	15.5 (11.0-21.5)	32.4 (28.6-36.3)
Lung disease	7.4 (6.1-9.0)	8.0 (6.1-10.4)	6.9 (4.6-10.2)
Heart disease	13.1 (10.9-15.8)	10.6 (8.5-13.1)	15.1 (12.0-18.9)
Stroke	4.3 (3.3-5.5)	3.6 (2.7-4.8)	4.8 (3.4-6.8)
Physical health			
Excellent, very good, or good	83.9 (82.0-85.7)	88.7 (85.2-91.5)	80.1 (76.3-83.3)
Fair or poor	16.1 (14.3-18.0)	11.3 (8.5-14.8)	19.9 (16.7-23.7)
Activities limited by disability	31.2 (29.1-33.3)	26.1 (22.7-29.9)	35.2 (31.1-39.5)

Abbreviations: BMI, body mass index (calculated as weight in kilograms divided by height in meters squared); NA, not applicable; SSDI, Social Security Disability Insurance.

^a^ Respondents were stratified by BMI of 27 or greater because that is the threshold above which respondents may be eligible for weight management medications with medical comorbidities (eg, hypertension, type 2 diabetes, hyperlipidemia).

### Interest in WMM

Overall, 35.1% (95% CI, 31.9%-38.4%) of adults ages 50 to 80 years were interested in using WMM, including 28.7% (95% CI, 23.4%-34.5%) of those with a BMI 27 to 29.9 and 59.1% (95% CI, 53.4%-64.5%) of those with a current BMI of 30 or greater (Table 2). Interest was most robustly associated with having used these medications in the past (adjusted odds ratio [aOR], 7.6 [95% CI, 4.4-13.0]) and with BMI of 30 or greater (aOR, 5.0 [95% CI, 3.5-7.3]). The odds of being interested in the use of WMM were higher for individuals who felt obesity was a chronic condition (aOR, 2.0 [95% CI, 1.1-3.6]) (Table 3).

**Table 2.** Interest in Taking WMM Stratified by Opinions About Obesity, Demographics, and Health Characteristics

Characteristic	% (95% CI)	
	Interested in using WMM^a^	Not interested in using WMM^b^
Total	35.1 (31.9-38.4)	64.9 (61.6-68.1)
Obesity is a lifestyle choice^c^^,^^d^		
Strongly or somewhat agree	30.9 (27.8-34.2)	69.1 (65.8-72.2)
Strongly or somewhat disagree	42.5 (36.1-49.1)	57.5 (50.9-63.9)
Obesity is a chronic condition^e^^,^^d^		
Strongly or somewhat agree	36.6 (33.1-40.4)	63.4 (59.6-66.9)
Strongly or somewhat disagree	21.0 (14.1-30.0)	79.0 (70.0-85.9)
Used any WMM^f^		
Yes	83.0 (76.1-88.2)	17.0 (11.8-23.9)
No	30.8 (27.6-34.2)	69.2 (65.8-72.4)
Age group, y^d^		
50-64	37.8 (34.1-41.7)	62.2 (58.3-65.9)
65-80	30.9 (26.5-35.7)	69.1 (64.3-73.5)
Gender^d^		
Male	31.9 (27.8-36.3)	68.1 (63.7-72.2)
Female	38.1 (34.5-41.7)	61.9 (58.3-65.5)
BMI^f^		
<27	18.7 (14.8-23.4)	81.3 (76.6-85.2)
27 to <30	28.7 (23.4-34.5)	71.3 (65.5-76.6)
≥30	59.1 (53.4-64.5)	40.9 (35.5-46.6)
Race and ethnicity		
Hispanic	44.2 (38.9-49.7)	55.8 (50.3-61.1)
Non-Hispanic Asian or Pacific Islander	34.7 (17.1-57.7)	65.3 (42.3-82.9)
Non-Hispanic Black	43.3 (36.6-50.2)	56.7 (49.8-63.4)
Non-Hispanic other race	29.9 (16.9-47.3)	70.1 (52.7-83.1)
Non-Hispanic, multiple races	37.8 (11.8-73.5)	62.2 (26.5-88.2)
Non-Hispanic White	32.5 (28.2-37.0)	67.5 (63.0-71.8)
Education^f^		
≤High school diploma	43.5 (37.7-49.5)	56.5 (50.5-62.3)
Some college	41.6 (38.0-45.3)	58.4 (54.7-62.0)
≥Bachelor’s degree	30.4 (26.2-35.0)	69.6 (65.0-73.8)
Annual household income, $^d^		
<30 000	45.1 (37.5-53.0)	54.9 (47.0-62.5)
30 000 to <60 000	35.8 (30.6-41.3)	64.2 (58.7-69.4)
60 000 to <100 000	35.0 (30.0-40.4)	65.0 (59.6-70.0)
≥100 000	30.9 (26.5-35.6)	69.1 (64.4-73.5)
Federal poverty level^d^		
≥100%	33.7 (30.9-36.6)	66.3 (63.4-69.1)
<100%	52.1 (38.8-65.2)	47.9 (34.8-61.2)
Employment status^f^		
Employed	35.9 (32.7-39.4)	64.1 (60.6-67.3)
Retired	29.5 (25.3-34.1)	70.5 (65.9-74.7)
Not working or receiving SSDI	47.0 (38.2-55.9)	53.0 (44.1-61.8)
Metropolitan area		
No	33.4 (25.7-42.1)	66.6 (57.9-74.3)
Yes	35.4 (32.2-38.8)	64.6 (61.2-67.8)
High blood pressure or hypertension^f^		
Yes	41.0 (36.5-45.8)	59.0 (54.2-63.5)
No	28.6 (25.3-32.2)	71.4 (67.8-74.7)
Diabetes or high blood glucose^f^		
Yes	45.4 (39.6-51.3)	54.6 (48.7-60.4)
No	31.7 (28.7-34.8)	68.3 (65.2-71.3)
Lung disease^d^		
Yes	47.0 (36.7-57.5)	53.0 (42.5-63.3)
No	34.0 (31.4-36.8)	66.0 (63.2-68.6)
Heart disease^g^		
Yes	42.0 (34.3-50.1)	58.0 (49.9-65.7)
No	34.1 (31.4-37.0)	65.9 (63.0-68.6)
Stroke^d^		
Yes	49.6 (37.9-61.4)	50.4 (38.6-62.1)
No	34.4 (31.6-37.3)	65.6 (62.7-68.4)
Physical health^f^		
Excellent, very good, or good	32.3 (29.2-35.6)	67.7 (64.4-70.8)
Fair or poor	49.4 (42.7-56.2)	50.6 (43.8-57.3)
Activities limited by disability^f^		
Yes	45.6 (38.8-52.7)	54.4 (47.3-61.2)
No	30.2 (27.6-32.9)	69.8 (67.1-72.4)

Abbreviations: BMI, body mass index (calculated as weight in kilograms divided by height in meters squared); SSDI, Social Security Disability Insurance; WMM, weight management medication.

^a^ Aggregated responses of strongly agree or somewhat agree to the statement: “I would be interested in taking prescription medications for weight management.”

^b^ Aggregated responses of strongly disagree or somewhat disagree to the statement: “I would be interested in taking prescription medications for weight management.”

^c^ Response to the statement: “Obesity is a lifestyle choice resulting from a person’s eating and exercise habits.”

^d^ Pearson χ^2^ *P* < .01.

^e^ Response to the statement: “Obesity is a chronic condition resulting from a combination of genetics, the food environment, medical conditions, and social factors.”

^f^ Pearson χ^2^ *P* < .001.

^g^ Pearson χ^2^ *P* < .05.

**Table 3.** Logistic Regression Analysis for Factors Associated With Opinions on WMM

Characteristic	Interest in using WMM^a^		Insurance should cover WMM^b^		Favor Medicare coverage of WMM^b^^,^^c^	
	aOR (95% CI)	*P* value	aOR (95% CI)	*P* value	aOR (95% CI)	*P* value
Obesity is a lifestyle choice^d^						
Strongly or somewhat agree	0.8 (0.5-1.1)	.11	0.3 (0.2-0.3)	<.001	0.5 (0.4-0.7)	<.001
Strongly or somewhat disagree	1 [Reference]		1 [Reference]		1 [Reference]	
Obesity is a chronic condition^e^						
Strongly or somewhat agree	2.0 (1.1-3.6)	.02	3.5 (2.3-5.3)	<.001	4.5 (2.7-7.4)	<.001
Strongly or somewhat disagree	1 [Reference]		1 [Reference]		1 [Reference]	
Taken any weight management medication						
Yes	7.6 (4.4-13.0)	<.001	3.5 (1.8-6.8)	<.001	1.2 (0.5-2.6)	.71
No	1 [Reference]		1 [Reference]		1 [Reference]	
Age group, y						
50-64	1 [Reference]	.85	1 [Reference]	.18	1 [Reference]	.21
65-80	1.0 (0.7-1.5)		0.7 (0.5-1.2)		0.8 (0.6-1.1)	
Gender						
Male	1 [Reference]	.04	1 [Reference]	.95	1 [Reference]	.002
Female	1.3 (1.0-1.6)		1.0 (0.7-1.3)		1.7 (1.2-2.3)	
BMI						
<27	1 [Reference]	NA	1 [Reference]	NA	1 [Reference]	NA
27-<30	1.5 (1.0-2.1)	.03	1.0 (0.6-1.7)	.99	0.8 (0.5-1.3)	.34
≥30	5.0 (3.5-7.3)	<.001	1.2 (0.9-1.8)	.27	1.1 (0.8-1.5)	.66
Race and ethnicity						
Hispanic	1.2 (0.9-1.7)	.30	0.6 (0.4-1.1)	.13	0.9 (0.6-1.5)	.77
Non-Hispanic Asian-Pacific Islander	1.4 (0.5-4.3)	.57	0.5 (0.2-1.1)	.09	0.8 (0.3-2.1)	.65
Non-Hispanic Black	1.1 (0.7-1.7)	.69	0.5 (0.3-0.7)	<.001	1.7 (1.1-2.6)	.009
Non-Hispanic other race	0.5 (0.2-1.0)	.05	0.5 (0.1-1.4)	.16	0.6 (0.3-1.1)	.12
Non-Hispanic, multiple races	0.8 (0.2-3.5)	.78	2.1 (0.6-7.5)	.24	1.5 (0.7-3.0)	.29
Non-Hispanic White	1 [Reference]	NA	1 [Reference]	NA	1 [Reference]	NA
Education						
≤High school diploma	1 [Reference]	NA	1 [Reference]	NA	1 [Reference]	NA
Some college	1.0 (0.7-1.3)	.81	1.1 (0.7-1.8)	.60	1.0 (0.6-1.7)	.94
≥Bachelor’s degree	0.7 (0.5-1.0)	.03	1.2 (0.7-2.2)	.44	0.9 (0.5-1.7)	.82
Federal poverty level						
≥100%	1 [Reference]	.28	1 [Reference]	.96	1 [Reference]	.30
<100%	1.4 (0.7-2.7)		1.0 (0.5-1.9)		0.6 (0.3-1.5)	
Current employment status						
Employed	1 [Reference]	NA	1 [Reference]	NA	1 [Reference]	NA
Retired	0.9 (0.7-1.1)	.32	1.6 (1.1-2.4)	.02	1.1 (0.8-1.6)	.39
Not working or receiving SSDI	0.9 (0.6-1.2)	.52	0.9 (0.5-1.6)	.72	1.6 (1.0-2.5)	.06
Metropolitan area						
No	1 [Reference]	.22	1 [Reference]	.06	1 [Reference]	.02
Yes	1.4 (0.8-2.3)		1.4 (1.0-1.9)		1.7 (1.1-2.6)	
High blood pressure or hypertension						
Yes	1.2 (0.9-1.6)	.22	1.4 (1.0-1.9)	.06	1.2 (0.9-1.6)	.14
No	1 [Reference]		1 [Reference]		1 [Reference]	
Diabetes or high blood glucose						
Yes	1.1 (0.8-1.5)	.45	1.2 (0.8-1.8)	.42	1.3 (0.9-1.8)	.17
No	1 [Reference]		1 [Reference]		1 [Reference]	
Lung disease						
Yes	1.3 (0.8-2.1)	.26	1.3 (0.6-2.9)	.57	2.1 (1.2-3.7)	.006
No	1 [Reference]		1 [Reference]		1 [Reference]	
Heart disease						
Yes	1.2 (0.8-1.7)	.33	1.2 (0.7-2.1)	.40	1.7 (1.0-2.7)	.04
No	1 [Reference]		1 [Reference]		1 [Reference]	
Stroke						
Yes	1.5 (0.9-2.5)	.10	1.0 (0.4-2.6)	.97	2.9 (1.6-5.2)	<.001
No	1 [Reference]		1 [Reference]		1 [Reference]	
Physical health						
Excellent, very good, or good	0.8 (0.6-1.2)	.30	0.9 (0.5-1.7)	.82	1.0 (0.6-1.7)	.88
Fair or poor	1 [Reference]		1 [Reference]		1 [Reference]	
Activities limited by disability						
Yes	1.2 (0.8-1.7)	.31	0.9 (0.6-1.3)	.58	1.0 (0.8-1.4)	.92
No	1 [Reference]		1 [Reference]		1 [Reference]	

Abbreviations: aOR, adjusted odds ratio; BMI, body mass index (calculated as weight in kilograms divided by height in meters squared); NA, not applicable; SSDI, Social Security Disability Insurance; WMM, weight management medication.

^a^ Response of strongly agree or somewhat agree to the statement: “I would be interested in taking prescription medications for weight management.”

^b^ Response to the question: “Do you think health insurance should cover the following treatments for overweight and obesity? –Prescription medication which has been FDA approved for weight loss.”

^c^ Response to the question: “Would you favor or oppose the following regarding FDA-approved prescription medication for weight management? –Require Medicare to cover these medications.”

^d^ Response to statement: “Obesity is a lifestyle choice resulting from a person’s eating and exercise habits.”

^e^ Response to statement: “Obesity is a chronic condition resulting from a combination of genetics, the food environment, medical conditions, and social factors.”

Adults ages 50 to 80 years of any BMI were more likely to have heard about the newer incretin mimetic class of WMM, including 60.7% (95% CI, 57.5%-63.9%) having heard of semaglutide (as *Ozempic*) and 18.3% (95% CI, 16.0%-20.7%) semaglutide (as *Wegovy*), compared with 13.1% (95% CI, 11.0%-15.6%) having heard of phentermine, 2.8% (95% CI, 2.1%-3.7%) having heard of liraglutide (as *Saxenda*), 2.8% (95% CI, 2.1%-3.7%) having heard of phentermine-topiramate (as *Qsymia*), and 3.3% (95% CI, 2.6%-4.2%) having heard of bupropion-naltrexone (as *Contrave*). Participants were more likely to report the news (57.6% [95% CI, 54.2%-60.9%]) and advertisements (53.1% [95% CI, 46.3%-59.8%]) as their source of information about WMM than their personal health care clinician (11.0% [95% CI, 8.4%-14.4%]).

Among participants with BMI of 27 or greater, few had used at least 1 FDA-approved WMM: 10.0% (95% CI, 7.0%-14.2%) had ever used phentermine; 1.5% (95% CI, 0.6%-3.3%), liraglutide (as *Saxenda*); 1.4% (95% CI, 0.5%-3.8%), bupropion-naltrexone; 1.3% (95% CI, 0.7%-2.4%), semaglutide (as *Wegovy*); and 0.6% (95% CI, 0.2%-2.2%), phentermine-topiramate. In comparison, 6.8% (95% CI, 4.7%-9.7%) reported ever having used semaglutide (as *Ozempic*) as approved for type 2 diabetes. Reported use of liraglutide, bupropion-naltrexone, and phentermine was higher among participants ages 50 to 80 years with commercial insurance compared with those without commercial insurance (eTable in Supplement 1).

### Perceptions About Coverage for WMM

Most adults ages 50 to 80 years (regardless of BMI) agreed that health insurance should cover WMM (2176 of 2625 respondents [83.2%]) (Table 4). When considering Medicare coverage specifically, 2097 of 2616 respondents (75.7%) favored coverage for medications and 829 of 2604 respondents (30.2%) approved of paying more for a Medicare premium to support coverage (Table 5). Participants who felt obesity was a chronic condition were more likely to favor insurance coverage (aOR, 3.5 [95% CI, 2.3-5.3]) and Medicare coverage (aOR, 4.5 [95% CI, 2.7-7.4]), while those who felt obesity was a lifestyle choice were less likely to favor insurance coverage for all insurance programs (aOR, 0.3 [95% CI, 0.1-0.4]) and for Medicare specifically (aOR, 0.5 [95% CI, 0.4-0.7]). Having been prescribed WMM was associated with supporting insurance coverage in general (aOR, 3.5 [95% CI, 1.8-6.8]) but not specifically Medicare coverage (Table 3).

**Table 4.** Opinion on Whether Prescription Weight Management Medication Should Be Covered by Insurance, Stratified by Opinions About Obesity, Demographics, and Health Characteristics

Characteristic	Prescription medication for weight loss should be covered by insurance, % (95% CI)^a^	
	Yes	No
Total	83.2 (81.3-85.0)	16.8 (15.0-18.7)
Obesity is a lifestyle choice^b^^,^^c^		
Strongly or somewhat agree	77.3 (74.5-79.9)	22.7 (20.1-25.5)
Strongly or somewhat disagree	93.5 (91.7-95.0)	6.5 (5.0-8.3)
Obesity is a chronic condition^d^^,^^c^		
Strongly or somewhat agree	85.7 (83.8-87.4)	14.3 (12.6-16.2)
Strongly or somewhat disagree	59.7 (49.3-69.4)	40.3 (30.6-50.7)
Used any WMM^c^		
Yes	95.6 (92.9-97.2)	4.4 (2.8-7.1)
No	82.1 (80.0-84.1)	17.9 (15.9-20.0)
Age group, y		
50-64	83.1 (79.6-86.2)	16.9 (13.8-20.4)
65-80	83.3 (79.7-86.4)	16.7 (13.6-20.3)
Gender		
Male	81.3 (78.6-83.6)	18.7 (16.4-21.4)
Female	85.0 (82.0-87.6)	15.0 (12.4-18.0)
BMI		
<27	81.1 (76.8-84.7)	18.9 (15.3-23.2)
27 to <30	83.7 (77.1-88.7)	16.3 (11.3-22.9)
≥30	87.1 (83.6-90.0)	12.9 (10.0-16.4)
Race/ethnicity		
Hispanic	80.4 (73.3-85.9)	19.6 (14.1-26.7)
Non-Hispanic Asian or Pacific Islander	73.3 (51.9-87.5)	26.7 (12.5-48.1)
Non-Hispanic Black	79.4 (75.2-83.1)	20.6 (16.9-24.8)
Non-Hispanic other race	76.5 (57.9-88.6)	23.5 (11.4-42.1)
Non-Hispanic, multiple races	92.8 (78.2-97.9)	7.2 (2.1-21.8)
Non-Hispanic White	85.0 (82.0-87.5)	15.0 (12.5-18.0)
Education		
≤High school diploma	78.9 (70.8-85.2)	21.1 (14.8-29.2)
Some college	84.2 (81.4-86.6)	15.8 (13.4-18.6)
≥Bachelor’s degree	83.6 (80.7-86.1)	16.4 (13.9-19.3)
Annual household income, $		
<30 000	82.7 (77.4-87.0)	17.3 (13.0-22.6)
30 000 to <60 000	86.1 (83.5-88.4)	13.9 (11.6-16.5)
60 000 to <100 000	80.1 (75.8-83.9)	19.9 (16.1-24.2)
≥100 000	83.9 (80.6-86.8)	16.1 (13.2-19.4)
Federal poverty level		
≥100%	83.2 (81.1-85.2)	16.8 (14.8-18.9)
<100%	83.1 (77.8-87.4)	16.9 (12.6-22.2)
Employment status		
Employed	82.5 (79.7-85.0)	17.5 (15.0-20.3)
Retired	85.4 (82.4-87.9)	14.6 (12.1-17.6)
Not working or receiving SSDI	80.3 (72.8-86.1)	19.7 (13.9-27.2)
Metropolitan area		
No	82.8 (76.9-87.4)	17.2 (12.6-23.1)
Yes	83.3 (81.2-85.2)	16.7 (14.8-18.8)
High blood pressure or hypertension^e^		
Yes	85.6 (82.8-87.9)	14.4 (12.1-17.2)
No	80.7 (77.6-83.4)	19.3 (16.6-22.4)
Diabetes or high blood glucose		
Yes	86.6 (82.2-90.1)	13.4 (9.9-17.8)
No	82.1 (79.7-84.3)	17.9 (15.7-20.3)
Lung disease		
Yes	84.5 (69.0-93.0)	15.5 (7.0-31.0)
No	83.1 (80.7-85.3)	16.9 (14.7-19.3)
Heart disease^e^		
Yes	88.7 (83.0-92.7)	11.3 (7.3-17.0)
No	82.5 (80.4-84.4)	17.5 (15.6-19.6)
Stroke		
Yes	86.3 (71.9-93.9)	13.7 (6.1-28.1)
No	83.2 (81.2-85.0)	16.8 (15.0-18.8)
Physical health		
Excellent, very good, or good	82.9 (80.7-85.0)	17.1 (15.0-19.3)
Fair or poor	84.6 (77.6-89.7)	15.4 (10.3-22.4)
Activities limited by disability		
Yes	84.0 (80.2-87.2)	16.0 (12.8-19.8)
No	82.9 (80.5-85.0)	17.1 (15.0-19.5)

Abbreviations: BMI, body mass index (calculated as weight in kilograms divided by height in meters squared); SSDI, Social Security Disability Insurance; WMM, weight management medication.

^a^ Response to the question: “Do you think health insurance should cover the following treatment for overweight and obesity? Prescription medication which has been FDA approved for weight loss.”

^b^ Response to statement: “Obesity is a lifestyle choice resulting from a person’s eating and exercise habits.”

^c^ Pearson χ^2^ *P* < .001.

^d^ Response to statement: “Obesity is a chronic condition resulting from a combination of genetics, the food environment, medical conditions, and social factors.”

^e^ Pearson χ^2^ *P* < .05.

**Table 5.** Opinions on Whether Prescription WMM Should Be Covered by Medicare, Stratified by Opinions About Obesity, Demographics, and Health Characteristics

Characteristic	Prescription medication for weight loss should be covered by Medicare, % (95% CI)^a^	
	Favor	Oppose
Total	75.7 (72.6-78.5)	24.3 (21.5-27.4)
Obesity is a lifestyle choice^b^^,^^c^		
Strongly or somewhat agree	70.0 (66.0-73.7)	30.0 (26.3-34.0)
Strongly or somewhat disagree	85.7 (81.8-88.9)	14.3 (11.1-18.2)
Obesity is a chronic condition^d^^,^^c^		
Strongly or somewhat agree	78.8 (75.5-81.8)	21.2 (18.2-24.5)
Strongly or somewhat disagree	47.3 (35.4-59.6)	52.7 (40.4-64.6)
Used any WMM		
Yes	85.8 (74.8-92.4)	14.2 (7.6-25.2)
No	74.8 (71.3-78.0)	25.2 (22.0-28.7)
Age group, y		
50-64	76.2 (72.0-80.0)	23.8 (20.0-28.0)
65-80	74.9 (71.5-78.0)	25.1 (22.0-28.5)
Gender^c^		
Male	69.9 (65.2-74.2)	30.1 (25.8-34.8)
Female	81.0 (78.1-83.6)	19.0 (16.4-21.9)
BMI		
<27	74.2 (68.0-79.6)	25.8 (20.4-32.0)
27 to <30	72.8 (67.0-77.9)	27.2 (22.1-33.0)
≥30	79.1 (73.7-83.7)	20.9 (16.3-26.3)
Race and ethnicity		
Hispanic	75.7 (70.7-80.1)	24.3 (19.9-29.3)
Non-Hispanic Asian or Pacific Islander	72.7 (51.5-87.0)	27.3 (13.0-48.5)
Non-Hispanic Black	85.6 (81.1-89.1)	14.4 (10.9-18.9)
Non-Hispanic other race	68.3 (56.6-78.1)	31.7 (21.9-43.4)
Non-Hispanic, multiple races	83.4 (73.1-90.2)	16.6 (9.8-26.9)
Non-Hispanic White	74.4 (70.1-78.3)	25.6 (21.7-29.9)
Education		
≤High school diploma	76.7 (67.8-83.7)	23.3 (16.3-32.2)
Some college	77.7 (74.2-80.9)	22.3 (19.1-25.8)
≥Bachelor’s degree	74.5 (70.7-78.0)	25.5 (22.0-29.3)
Annual household income, $^e^		
<30 000	77.5 (70.3-83.4)	22.5 (16.6-29.7)
30 000 to <60 000	82.9 (79.5-85.8)	17.1 (14.2-20.5)
60 000 to <100 000	69.6 (63.4-75.2)	30.4 (24.8-36.6)
≥100 000	75.2 (70.8-79.2)	24.8 (20.8-29.2)
Federal poverty level		
≥100%	75.6 (72.4-78.5)	24.4 (21.5-27.6)
<100%	77.1 (63.9-86.5)	22.9 (13.5-36.1)
Employment status		
Employed	73.8 (68.7-78.3)	26.2 (21.7-31.3)
Retired	76.7 (73.0-80.0)	23.3 (20.0-27.0)
Not working or receiving SSDI	81.3 (74.4-86.6)	18.7 (13.4-25.6)
Metropolitan area		
No	70.8 (64.0-76.8)	29.2 (23.2-36.0)
Yes	76.6 (72.8-80.0)	23.4 (20.0-27.2)
High blood pressure or hypertension		
Yes	77.8 (74.5-80.8)	22.2 (19.2-25.5)
No	73.4 (68.9-77.4)	26.6 (22.6-31.1)
Diabetes or high blood glucose^f^		
Yes	80.3 (75.4-84.4)	19.7 (15.6-24.6)
No	74.2 (70.5-77.6)	25.8 (22.4-29.5)
Lung disease^c^		
Yes	85.9 (81.9-89.2)	14.1 (10.8-18.1)
No	74.9 (71.3-78.2)	25.1 (21.8-28.7)
Heart disease^f^		
Yes	83.4 (75.9-88.9)	16.6 (11.1-24.1)
No	74.6 (71.2-77.8)	25.4 (22.2-28.8)
Stroke^c^		
Yes	90.2 (84.3-94.0)	9.8 (6.0-15.7)
No	75.1 (71.7-78.2)	24.9 (21.8-28.3)
Physical health		
Excellent, very good, or good	74.9 (71.3-78.1)	25.1 (21.9-28.7)
Fair or poor	80.0 (74.0-84.8)	20.0 (15.2-26.0)
Activities limited by disability^f^		
Yes	80.0 (76.5-83.2)	20.0 (16.8-23.5)
No	73.7 (69.6-77.4)	26.3 (22.6-30.4)

Abbreviations: BMI, body mass index (calculated as weight in kilograms divided by height in meters squared); SSDI, Social Security Disability Insurance; WMM, weight management medication.

^a^ Response to the question: “Would you favor or oppose the following regarding FDA-approved prescription medication for weight management? Require Medicare to cover these medications.”

^b^ Response to statement: “Obesity is a lifestyle choice resulting from a person’s eating and exercise habits.”

^c^ Pearson χ^2^ *P* < .001.

^d^ Response to statement: “Obesity is a chronic condition resulting from a combination of genetics, the food environment, medical conditions, and social factors.”

^e^ Pearson χ^2^ *P* < .01.

^f^ Pearson χ^2^ *P* < .05.

## Discussion

In this cross-sectional survey study, most older adults favored commercial insurance and Medicare coverage of WMMs (83.2% and 75.7%, respectively), but fewer (30.2%) favored higher Medicare premiums to support this coverage. Similar numbers of adults ages 18 years and older favored insurance coverage for WMM in a recent national poll.^22^ A recent Congressional Budget Office report estimated $35 billion in increased federal spending over 8 years, assuming 14% use of incretin mimetics for weight management among eligible Medicare beneficiaries.^15^ A recent US Senate Committee report estimated increased Medicare spending of $96.1 billion annually based on 50% uptake among beneficiaries with obesity.^23^ Our study found that 59.1% of older adults with obesity (BMI ≥30) and 35.1% of all older adults would be interested in using WMMs. Furthermore, participants who had previously been prescribed WMMs were more likely to be interested in future use. These results suggest that the Congressional Budget Office report may be underestimating the interest and uptake of incretin mimetics for weight management. While our results show high interest in initiating WMMs, other surveys have suggested decreased interest based on the injection route of administration.^24^ Recent studies suggest variable adherence and continuation of incretin mimetics based on the type of WMM, indication (type 2 diabetes or weight management), effectiveness for weight loss, cost of medication, and access to continued insurance coverage.^25,26,27,28^ It remains unknown what percentage of older adults would initiate and continue incretin mimetic treatment if covered, and how this would affect projected costs.

A cost-effective population health approach to treatment for the half of all US adults affected by obesity would reserve the costliest medications for people with more severe disease or those who failed to benefit from less expensive, effective treatments. Such an approach should also balance potential harms of substantial weight loss, such as sarcopenia, with potential benefits for older patients.^29^ While incretin mimetics, like semaglutide and tirzepatide, are effective at achieving weight loss of 15% or more, list prices currently exceed $1000 per month, and direct-to-consumer noninsurance prices for tirzepatide 5 mg are currently $549 per month.^30,31^ However, population-level studies suggest that smaller amounts of weight loss can improve population-level health and can be accomplished by selective use of less expensive WMM, such as phentermine-topiramate, bupropion-naltrexone, and phentermine. At current list prices, the Institute for Clinical and Economic Review found only phentermine-topiramate to be cost-effective in additional to lifestyle treatment for weight management.^32^ List prices for incretin mimetics may decrease over time and do not reflect the true cost to Medicare insurers after pharmacy benefit management strategies, including discount negotiations with pharmaceutical manufacturers, rebate pass-throughs, step therapy, and other prior authorization strategies.^33^ While a generic liraglutide 3.0 mg may be available as soon as 2026, the typical weight loss effectiveness of this incretin mimetic is significantly lower than semaglutide and tirzepatide.^34^ In addition, lifestyle change programs, like the National Diabetes Prevention Program, are effective at preventing complications of obesity at lower cost, and bariatric (metabolic) surgery is beneficial at reducing incident cancer and diabetes and lowering mortality.^35^

In this study, adults ages 50 to 80 years were unlikely to have heard of less expensive yet effective medications to treat obesity, and less than 2% had used any non–incretin mimetic therapy, yet most information about these medications were coming from news and advertisements, not physicians. At least one popular social media site has banned advertisement of obesity medications,^36^ and direct-to-consumer advertising remains controversial in the US. To meet the vast needs for obesity care for older adults in the US, primary care clinicians and their teams must receive adequate education and resources to recommend non–incretin mimetic medications to appropriate patients with an FDA indication for treatment, such as obesity or overweight with comorbidities. In addition, more research is needed to guide cost-effective allocation of these medications to those who will benefit most.^37^ Finally, as participants in this poll less frequently favored paying more for coverage of medications, focusing prescribing for patients most likely to have expensive complications of obesity and using the negotiating power of Medicare to lower costs of expensive medications are strategies that should be considered.^38^ The US Department of Health and Human Services plans price negotiation under the Inflation Reduction Act for semaglutide in 2025, with negotiated prices taking effect in 2027.^39^ As we were unable to ask additional questions about specific premium, deductible, and copayment increases that would be favorable to adults ages 50 to 80 years, further research in this area may expand on our results.

### Limitations

Our study had several limitations. First, we surveyed a nationally representative sample of adults ages 50 to 80 years, but our findings may not be representative of all older adults. Second, respondents may be more or less likely than nonrespondents to engage in weight management treatment or have different awareness of WMM. Third, we oversampled for Black and Hispanic respondents to increase the reliability of estimands for these groups, contributing to an overall lower completion rate. Fourth, we used self-reported weight and height to calculate BMI, and it is not possible to confirm the accuracy of this information.

## Conclusions

In this survey study of older adults, participants overwhelmingly supported insurance, specifically Medicare, coverage of WMM. These results should inform decisions around inclusion of this class of medications in the Medicare program, as well as utilization and cost control policies to improve population health in a cost-effective manner.

## Notes

Supplement 1. **eTable.** Weight Management Medication Use by Type of Insurance

Supplement 2.
Data Sharing Statement
