# Supplementary material for: Older Adults’ Views on Insurance Coverage for Weight Management Medications
Source: JAMA Netw Open. 2025 Mar 26;8(3):e252008. doi: 10.1001/jamanetworkopen.2025.2008 (PMC11947835; doi:10.1001/jamanetworkopen.2025.2008)
Supplement: Supplement 1. — eTable. Weight Management Medication Use by Type of Insurance [file jamanetwopen-e252008-s001.pdf]

Supplemental Online Content

Oshman L, Kirch M, Solway E, et al. Older adults' views on insurance coverage for weight management medications. *JAMA Netw Open*. 2025;8(3):e252008. doi:10.1001/jamanetworkopen.2025.2008

eTable. Weight Management Medication Use by Type of Insurance

This supplemental material has been provided by the authors to give readers additional information about their work.

© 2025 Oshman L et al. *JAMA Network Open*.

eTable 1: Weight management medication use by type of insurance

Type of weight management medication used	Among those with public insurance only[a] % (95% CI)	Among those with any commercial insurance[b] % (95% CI)
Wegovy (semaglutide)	1.2 (0.6, 2.4)	0.8 (0.4, 1.7)
Ozempic (semaglutide)	5.2 (2.8, 9.6)	5.5 (3.9, 7.6)
Saxenda (liraglutide)[c]	0.2 (0.1, 0.8)	1.3 (0.5, 3.0)
Qsymia (phentermine-topiramate)	0.8 (0.2, 3.2)	0.2 (0.0, 0.7)
Contrave (bupropion-naltrexone)[d]	0.0 (0.0, 0.1)	1.3 (0.5, 3.6)
Phentermine[c]	4.7 (3.1, 7.2)	7.3 (5.1, 10.5)

Pearson, *p<.05, **p<.01, ***p<.001

[a]Public insurance only indicates one of the following sources of insurance with no supplemental plans: traditional Medicare, Medicare Advantage, Medicaid, VA/CHAMPA, military health care (TRICARE).

[b]Commercial insurance includes employer-sponsored insurance, retiree health plans, supplemental Medicare plans, and individual plans through the marketplace.

[c]Pearson Chi^2 $p<0.05$

[d]Pearson Chi^2 $p<0.001$

© 2025 Oshman L et al. *JAMA Network Open*.
