# Supplementary material for: Older Adults’ Views on Insurance Coverage for Weight Management Medications
Source: JAMA Netw Open. 2025 Mar 26;8(3):e252008. doi: 10.1001/jamanetworkopen.2025.2008 (PMC11947835; doi:10.1001/jamanetworkopen.2025.2008)
Supplement: Supplement 2. — Data Sharing Statement [file jamanetwopen-e252008-s002.pdf]

Data Sharing Statement

Oshman. Older Adults' Views on Insurance Coverage for Weight Management Medications. *JAMA Netw Open*. Published March 26, 2025. doi:10.1001/jamanetworkopen.2025.2008

Data
Data available: Yes
Data types: Deidentified participant data
How to access data: This is all available at the National Poll on Healthy Aging. https://www.healthyagingpoll.org/reports-more/data --> https://www.openicpsr.org/openicpsr/search/studies?start=0&ARCHIVE=openicpsr&sort=score%20desc%2CDATEUPDATED%20desc&rows=25&q=national%20poll%20on%20healthy%20aging
When available: With publication

Supporting Documents
Document types: None

Additional Information
Who can access the data: Anyone who can access the website
Types of analyses: Per the site: National Poll on Healthy Aging Data Available for Free Public Use The National Poll on Healthy Aging publishes its data for public use to help advance research on aging and health. Data files are now available for download through the National Archive of Computerized Data on Aging (NACDA)'s Open Aging Repository, a free self-publishing option for gerontological researchers to share their work. The data below can be downloaded in Stata, SAS, and SPSS formats. Additional data will be archived on an ongoing basis.
Mechanisms of data availability: see above
